# Socioeconomic gradient in consumption of whole fruit and 100% fruit juice among US children and adults

**DOI:** 10.1186/1475-2891-14-3

**Published:** 2015-01-05

**Authors:** Adam Drewnowski, Colin D Rehm

**Affiliations:** Department of Epidemiology and Center for Public Health Nutrition, University of Washington, Box 353410, Seattle, WA 98195 USA

**Keywords:** Fruit, Fruit juice, Diet quality, Dietary surveillance, Socioeconomic factors, Child nutrition, Adult nutrition

## Abstract

**Background:**

The consumption of fruit is generally associated with better health, but also higher socioeconomic status (SES). Most previous studies evaluating consumption of fruits have not separated 100% fruit juice and whole fruit, which may conceal interesting patterns in consumption.

**Objective:**

To estimate demographic and socioeconomic correlates of whole fruit versus 100% juice consumption among children and adults in the United States.

**Design:**

Secondary analyses of two cycles of the nationally representative National Health and Nutrition Examination Survey (NHANES) from 2007–2010, by gender, age group, race/ethnicity and SES among 16,628 children and adults.

**Results:**

Total fruit consumption (population average of 1.06 cup equivalents/d) fell far short of national goals. Overall, whole fruit provided about 65% of total fruit, while 100% juice provided the remainder. Whereas 100% juice consumption was highest among children and declined sharply with age, whole fruit consumption was highest among older adults. Total fruit and whole fruit consumption was generally higher among those with higher incomes or more education. By contrast, the highest 100% juice consumption was found among children, racial/ethnic minorities and lower-income groups.

**Conclusions:**

Consumption patterns for whole fruit versus 100% fruit juice showed different gradients by race/ethnicity, education, and income. The advice to replace 100% juice with whole fruit may pose a challenge for the economically disadvantaged and some minority groups, whose fruit consumption falls short of national goals.

## Introduction

The 2010 Dietary Guidelines for Americans (DGAs) recommends increasing consumption of fruit [1]. However, in all but the youngest children, total fruit consumption in the United States falls short of recommended levels [1–3]. The 2010 DGAs specify that 100% fruit juice is an important component of total fruit consumption, but does recommend limiting its consumption given its lack of dietary fiber and potential for excess consumption. The American Academy for Pediatrics recommends capping fruit juice consumption at 4–6 fluid ounces per day (0.5-0.75 cups/day) for children 1-6y and 8–12 fluid ounces per day (1–1.5 cups/day) for those 7-18y [4]. Fruit, including whole and 100% fruit juice, contribute substantial amounts of vitamin C and potassium. Whole fruit is generally dense in dietary fiber, and many fruit juices are fortified with vitamin D and/or calcium, all of which were identified in the DGAs as nutrients of concern given low levels of consumption [1, 5, 6]. Overall, consumption of total fruit falls far below recommended levels; however, some socio-demographic groups are more likely to consume inadequate amounts of total fruit.

This shortfall in fruit consumption appears to be highest among lower socioeconomic groups, both in the US [7] and elsewhere [8–11]. Fruit consumption is lower in lower-income neighborhoods and among some racial/ethnic minority groups [12, 13]. Evidence from studies of dietary patterns among adults suggests that the consumption of fruit follows a socioeconomic gradient [14–17]. Fruits represent a large and heterogeneous food group, with a wide variety of tastes, textures, culinary uses, and prices. Some have suggested that the determinants and profiles of fruit consumption ought to be disaggregated by type of fruit (e.g., whole vs. 100% fruit juice) [18]. However, most descriptive work characterizing dietary intakes of the population and intervention studies do not disaggregate results by type of fruit.

Beyond this, few studies of either children or adults have focused on the socioeconomic correlates of fruit consumption alone. In particular, the potential presence of a socioeconomic gradient in the consumption of whole fruit versus 100% fruit juice has not been examined [18]. Summaries of population-level dietary patterns for whole and 100% fruit juice provides important information on the potential challenges and implications of calls from the 2010 Dietary Guidelines for Americans [1] and the American Academy of Pediatrics [4] to replace 100% juice with whole fruit. To address gaps in the availability of descriptive data on fruit, whole fruit and 100% fruit juice consumption, we conducted a population-based cross-sectional study using data from the National Health and Nutrition Examination Survey.

## Methods

### NHANES participants

Data analyses were based on two consecutive cycles of the National Health and Nutrition Examination Survey (NHANES) from 2007–2008 and 2009–2010. The total population sample was 16,628 children, adolescents and adults age ≥ 4y. All data on population sub-groups came from the demographic questionnaire. Population subgroups were based on age group (4-13y, 14-19y, 20-50y, ≥51y); gender; race/ethnicity (non-Hispanic white, non-Hispanic black, Mexican-American, and other Hispanic), and family income-to-poverty ratio (IPR: <1.3, 1.3-3.49 and ≥3.5). The family income-to-poverty ratio is a ratio of a family’s income to the federal poverty level and is often used to determine eligibility for government assistance programs. In 2010, the federal poverty level was $22,050/yr for a family of 4; therefore a family earning $35,000/yr would have a family income-to-poverty ratio of 1.59. Education was used as an additional measure of socioeconomic status for adults ≥ 25y (less than high school, high school/equivalent, some college and college degree or higher). Family income-to-poverty ratio and education were used as measures of SES, in the current study as they measure the two most important components of SES in the US (income and educational attainment) and are collected in most health/nutrition studies. NHANES data are publicly available and are considered exempt from human subjects review by the University of Washington.

### Dietary recall data

The NHANES 24-h recall used a multi-pass method, where respondents reported the types and amounts of all food and beverages consumed in the preceding 24-hours, from midnight to midnight. For children aged 4-5y the dietary recall was completed entirely by a proxy respondent. For children aged 6-11y, the child was the primary respondent, but a proxy respondent was present and able to assist. For children aged 12-19y, the child/adolescent was the primary source of dietary recall information, but could be assisted by an adult who had knowledge of their diet [19]. The NHANES database included 2 dietary recalls. All participants completed the first dietary recall, which was completed in-person at the Mobile Examination Center with a trained interviewer. The second was completed over the telephone some days later by 87% of participants.

### Whole fruit versus 100% juice consumption

Usual intakes of total fruit, whole fruit, and 100% fruit juice were assessed for the entire population and for population subgroups. Whole fruit and 100% fruit juice were differentiated using information on the relative content of whole vs. 100% fruit juice per 100 gram for each food or beverage item in the 2003–2004 USDA Center for Nutrition Policy and Promotion Healthy Eating Index support files [20].

Outcome variables were expressed as: cup equivalents of total fruit, whole fruit, and 100% fruit juice. A cup equivalent of fruit corresponded to 1 small apple, 1 large banana, ½ cup of dried fruit, or 1 cup of 100% juice (i.e., 8 fluid ounces). Fruit cup equivalents were obtained from the USDA MyPyramid Equivalents Database (MPED), which includes information on total fruit consumption corresponding to previous cycles of NHANES [21]. Because MPED data were not available corresponding to more recent cycles of NHANES, we used the MPED addendum database from the Center for Policy and Promotion [22]. Analyses were conducted prior to the September 2013 release of the Food Patterns Equivalent Database, containing updated groups related to 2010 Dietary Guidelines for Americans.

### Statistical analysis

The National Cancer Institute (NCI) Method was used to characterize the usual intake distributions of total fruit, whole fruit, and 100% fruit juice from NHANES data. Episodic models were used to evaluate the usual intake distribution of fruit, whole fruit and 100% fruit juice, as no more than 90% of participants for any population sub-group consumed any fruit on their recall day. Additional covariates were included in the model to account for whether the recall data were from a weekday or weekend and whether it was the first or second recall. The episodic model [23] incorporates both the probability of consumption and the amount in estimating the usual intake distribution [24, 25]. In order to account for the complex survey design of NHANES data, balanced repeated replication (BRR) weights were constructed using WesVar software [26] and a Fay’s adjustment of 0.7. A total of 32 BRR runs were repeated for each analysis, making the results representative of the US population.

The outcomes of interest were the means and median cup equivalents for total fruit, whole fruit and 100% fruit juice. Another outcome was the proportion of each population subgroup whose fruit intake was below specified thresholds. Specified thresholds were 1.5 and 2 cup equivalents/day, which were selected as cut-points as they correspond to recommendations made by the USDA MyPlate program for most gender and age group strata being evaluated (i.e., 1.5 cups/d are recommended for girls 9-18y, while women 19-30y should consume 2 cups/d) [27]. Because the NCI Method uses a random seed in running the models, values that would otherwise be expected to sum together may not do so perfectly (i.e., repeated runs of the same model can result in differences of 1-2% between runs).

The percent contribution of whole fruit versus 100% fruit juice to total fruit consumption by population subgroups was estimated by dividing the whole/juice value by the total fruit value. To ensure consistency, estimates of population proportion used the sum of whole fruit and fruit juice rather than the estimated total fruit value as presented in tables. The population proportion is the percent total fruit from whole/juices sources at population-level. This measure can be interpreted as a ratio of the means, rather than a mean of the ratios, and is best suited for examinations of population-level dietary habits [28, 29].

T-tests were used to test differences in the mean intake level and proportion in each sub-group whose consumption was below pre-specified levels compared to a relevant reference group. The reference groups used were non-Hispanic whites, family income-to-poverty ratio ≥ 3.5 and those with a college degree or higher. Secondary analyses were conducted to evaluate the impact of adjustment for socio-demographic covariates in analyses of family income, education and race/ethnicity. Analyses of family income and education adjusted for age group (not included in age-specific analyses), gender and race/ethnicity. Analyses of race/ethnicity adjusted for age group, gender and family income, as data on education was only available for adults. If adjustment for these covariates appreciably changed conclusions, the impact of this is noted in the Results section. All analysis used SAS 9.3 [30] and estimates of the usual intake distribution used code and methods adapted from the NCI and Centers for Disease Control [23, 31].

## Results

Table 1 shows mean intakes for total fruit, whole fruit and 100% fruit juice by age group, income to poverty ratio and race/ethnicity. Total fruit cup equivalents for the population ≥ 4y were 1.06, far short of the recommended amounts: 1.5 to 2.0 cup equivalents per day, depending on age.

**Table 1:** **Mean servings of total fruit, whole fruit and 100% fruit juice by age group, family income-to-poverty ratio, and race/ethnicity, NHANES 2007-2010**

	n	Total fruit (SE)	Whole fruit (SE)	Fruit juice (SE)
Total	16,628	1.06 (0.02)	0.68 (0.01)	0.37 (0.01)
Gender				
Male (ref)	8,282	1.13 (0.02)	0.71 (0.02)	0.43 (0.01)
Female	8,346	1.00 (0.02)***	0.66 (0.02)	0.33 (0.01)***
Age group (y)				
4-13	3,612	1.20 (0.03)***	0.75 (0.03)	0.47 (0.02)***
14-19	1,834	1.06 (0.05)	0.62 (0.04)***	0.45 (0.03)***
20-50	5,793	0.99 (0.02)**	0.61 (0.02)***	0.39 (0.02)***
≥51(ref)	5,389	1.08 (0.01)	0.79 (0.01)	0.29 (0.01)
Income-to-poverty ratio^a^				
<1.3	5,509	1.00 (0.03)*	0.55 (0.02)***	0.42 (0.02)*
1.3-3.49	5,652	0.98 (0.02)**	0.63 (0.02)***	0.35 (0.02)
≥3.5 (ref)	4,013	1.10 (0.03)	0.77 (0.02)	0.35 (0.01)
Race/ethnicity				
Non-Hispanic white (ref)	7,102	1.00 (0.02)	0.69 (0.02)	0.31 (0.01)
Non-Hispanic black	3,414	1.10 (0.02)***	0.53 (0.02)***	0.58 (0.02)***
Mexican-American	3,436	1.19 (0.03)***	0.73 (0.03)	0.44 (0.02)***
Other Hispanic	1,843	1.31 (0.04)***	0.68 (0.02)	0.56 (0.04)***

Reference group identified in parentheses. Asterisks indicate significant difference in mean compared to the reference group (***p < 0.001; **0.001 < p < 0.01; *0.01 < p < 0.05).

^a^Family income-to-poverty is the ratio of family income to the federal poverty level. In 2010, the federal poverty level was $22,050/yr for a family of 4.

Mean levels of total fruit consumption by age exhibited a bimodal distribution. Young children (4-13y) and adults ≥ 51y consumed more total fruit than either adolescents or younger adults. Men consumed more total fruit than women, a finding likely driven by higher energy intakes among men. In analyses unadjusted for socio-demographic covariates, total fruit consumption increased with family income-to-poverty ratio and was higher for Mexican-Americans and other Hispanics than for non-Hispanic whites and non-Hispanic blacks. Results were unchanged upon adjustment for age group, gender, race/ethnicity (for income results) and income (for race/ethnicity results).

On average, total fruit consumption was composed of 0.68 cup equivalents of whole fruit and 0.37 cup equivalents of 100% fruit juice. However, distinct gradients were observed in whole fruit versus 100% fruit juice consumption by age group, race/ethnicity and family income. Older adults consumed the most whole fruits (0.77 cup equiv/d), while the consumption of 100% juice declined sharply with age, from 0.45 cup equiv/d at age 4-13y to 0.29 cup equiv/d for those ages ≥ 50y.

Whole fruit consumption was lower among individuals living in lower income households (p < 0.001), and was lowest in the non-Hispanic black population (0.53 cup equiv/d, p < 0.001) as compared to the other race/ethnicity groups. Conversely, 100% fruit juice consumption was higher at lower incomes and was higher among Mexican-American and non-Hispanic black as compared to non-Hispanic whites. Results for whole fruit were not impacted upon adjustment for age group, gender, race/ethnicity (for income analyses) and income (for race/ethnicity analyses). However, adjustment for age group, gender and race/ethnicity did result in non-significant differences in fruit juice consumption between the lowest and highest income groups. This suggests that the strong relationship between race/ethnicity and fruit juice consumption was driving the unadjusted association by income.

Table 2 shows the proportion of the population consuming less than the recommended amount of total fruit. The thresholds used were 1.5 and 2.0 cup equivalents. On average, 75% of the population failed to meet the 1.5 cup equivalents threshold, whereas 87% failed to meet the 2.0 cup threshold. Failing to meet the total fruit thresholds was associated with being female, mid-to-lower-income and non-Hispanic black. This socio-demographic gradient was observed for all age groups.

**Figure 1:**
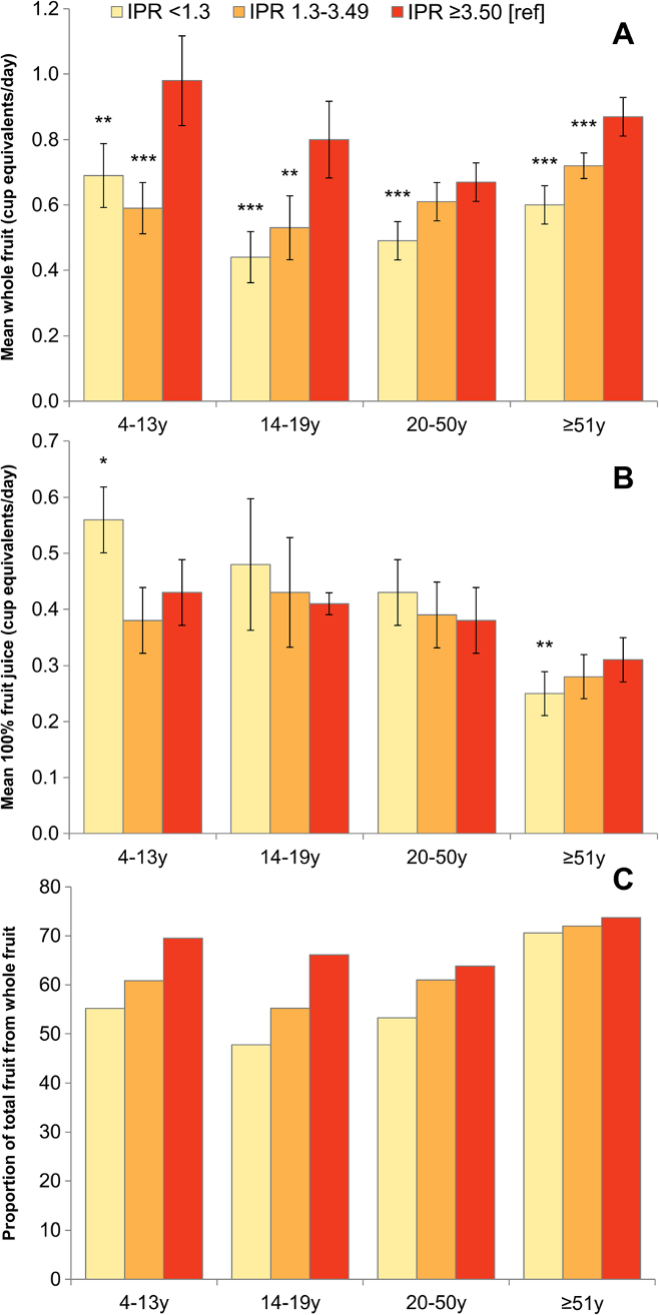
**Amounts of whole fruit and fruit juice (in cup equivalents) and proportion of total fruit from whole fruit by age group. Panel A**: whole fruit; **Panel B**: 100% fruit juice; **Panel C**: Proportion of total fruit from whole fruit. NHANES 2007–10. Error bars are 95% confidence intervals and asterisks indicate significant difference in mean compared to the highest income group (***p < 0.001; **0.001 < p < 0.01; *0.01 < p < 0.05). Significance testing for proportions not conducted.

Figure 1 shows the social gradient by family income-to-poverty ratio in consumption of whole fruit versus 100% fruit juice by age group. Panel A and B show the mean intakes of whole and 100% fruit juice, respectively, while Panel C shows the proportion of total fruit from whole fruit. Among all age groups, individuals with the lowest income consumed significantly less whole fruit than higher income individuals (p < 0.01). Among children 4-13y lower-income children consumed significantly more 100% fruit juice than higher-income children. Interestingly, among older adults the opposite was true, with lower income adults consuming significantly less 100% fruit juice than higher income adults, though absolute intakes of fruit juice were lower in this population sub-group. Adjustment for gender and race/ethnicity did not alter the observed age-specific associations between family income and whole fruit and 100% fruit juice consumption. Overall, whole fruit accounted for about 65% of total fruit consumption. The proportion of total fruit cup equivalents from 100% fruit juice ranged from 27-42%, depending on age. Consuming a greater proportion of total fruit from whole fruit was associated with being female, non-Hispanic white, and living in a higher income household (data for gender and race not shown, data for income shown in Figure 1). By contrast, consuming a higher proportion of total fruit from 100% fruit juice was associated with being non-Hispanic black and having a lower income (data for race not shown, data for income shown in Figure 1).

**Table 2:** **Proportion of population consuming less than specified threshold amounts of total fruit, NHANES 2007-2010**

	Age ≥ 4		Age 4-13y		Age 14-19y		Age 20-50y		Age ≥ 51y	
	1.5 servings	2.0 servings	1.5 servings	2.0 servings	1.5 servings	2.0 servings	1.5 servings	2.0 servings	1.5 servings	2.0 servings
Total	76.0 (0.7)	87.3 (0.5)	71.9 (1.6)	88.3 (1.1)	76.6 (2.2)	87.8 (1.7)	77.7 (0.9)	87.5 (0.7)	75.0 (0.6)	87.5 (0.5)
Gender										
Male (ref)	73.2 (0.7)	84.7 (0.6)	72.7 (1.5)	87.4 (1.1)	73.9 (1.5)	84.7 (1.2)	73.1 (1.0)	83.8 (0.8)	74.0 (1.2)	85.7 (0.9)
Female	78.9 (0.9)***	90.0 (0.6)****	71.2 (2.7)	90.0 (1.9)	87.8 (3.3)***	96.4 (1.8)***	81.6 (1.1)***	90.4 (0.8)***	76.0 (1.0)	89.2 (0.7)***
Income-to-poverty ratio^a^										
<1.3	77.9 (1.4)	89.1 (1.0)*	68.9 (3.1)	88.0 (2.1)	87.6 (3.5)*	97.5 (1.5)**	79.0 (1.7)	88.2 (1.2)	81.7 (1.4)***	91.2 (1.0)***
1.31-3.49	78.3 (1.0)*	88.4 (0.6)	83.1 (2.8)***	93.9 (1.5)***	78.1 (2.2)	86.4 (1.8)	77.4 (1.3)	86.7 (1.0)	79.3 (1.4)***	90.2 (0.9)***
≥3.5 (ref)	74.3 (1.2)	86.3 (1.0)	64.2 (3.8)	83.8 (2.6)	73.5 (5.2)	86.5 (4.3)	78.4 (1.6)	88.0 (1.1)	70.9 (1.1)	84.7 (0.8)
Race/ethnicity										
Non-Hispanic white (ref)	78.1 (0.7)	88.5 (0.6)	75.6 (2.2)	90.2 (1.5)	82.4 (2.9)	90.7 (2.1)	80.3 (1.0)	88.9 (0.8)	75.2 (0.7)	87.6 (0.5)
Non-Hispanic black	75.1 (1.1)*	87.5 (0.8)	73.5 (3.5)	92.5 (2.1)	72.6 (3.2)*	86.8 (2.2)	79.4 (1.7)	89.4 (1.2)	70.9 (1.6)**	83.5 (1.3)**
Mexican-American	70.2 (1.2)***	83.8 (0.9)***	64.0 (2.6)***	81.6 (2.4)**	74.6 (3.3)	85.6 (2.5)	68.8 (1.9)***	82.2 (1.3)***	79.4 (2.0)*	90.5 (1.3)*
Other Hispanic	65.3 (1.8)***	81.3 (1.5)***	67.8 (4.0)*	86.8 (2.6)	65.6 (6.0)**	80.6 (3.9)**	61.5 (3.2)***	76.8 (2.7)***	73.3 (3.5)	89.8 (2.3)

Asterisks indicate significant difference in mean compared to the reference group (***p < 0.001; **0.001 < p < 0.01; *0.01 < p < 0.05).

^a^Family income-to-poverty is the ratio of family income to the federal poverty level. In 2010, the federal poverty level was $22,050/yr for a family of 4.

Table 3 shows the patterns of total fruit, whole fruit, and 100% juice consumption for adults ages ≥25y, stratified by education level. First, the greatest total fruit intakes were associated with the most education. Second, the highest levels of whole fruit intakes were also associated with higher education when compared to other groups. In contrast, the consumption of 100% fruit juice did not show a strong positive gradient by education; only among older adults was 100% fruit juice consumption linked to higher educational status, similar to results observed for family income (see Figure 1, Panel B). Adjustment for age group, gender and race/ethnicity did not alter the observed associations between education and total fruit, whole fruit or fruit juice consumption.

**Table 3:** **Total fruit, whole fruit and 100% juice consumption (servings) for adults ≥25y by education, NHANES 2007-2010**

	Age ≥ 25		Age 25-50y		Age ≥ 51y	
	Mean (SE)	Median (SE)	Mean (SE)	Median (SE)	Mean (SE)	Median (SE)
Total fruit						
<High school	0.87 (0.03)***	0.66 (0.03)	0.81 (0.05)***	0.58 (0.05)	1.01 (0.03)***	0.85 (0.03)
High school/equivalent	0.90 (0.03) ***	0.71 (0.03)	0.81 (0.04)***	0.56 (0.05)	0.93 (0.03)***	0.75 (0.03)
Some college	1.01 (0.02) ***	0.79 (0.02)	1.01 (0.03)	0.71 (0.03)	1.04 (0.03)***	0.91 (0.04)
≥College (ref)	1.22 (0.03)	1.02 (0.04)	1.12 (0.06)	0.90 (0.05)	1.37 (0.03)	1.21 (0.03)
Whole fruit						
<High school	0.58 (0.03)***	0.37 (0.02)	0.46 (0.04)***	0.26 (0.03)	0.71 (0.03)***	0.52 (0.02)
High school/equivalent	0.60 (0.02)***	0.44 (0.02)	0.51 (0.04)***	0.33 (0.04)	0.71 (0.02)***	0.58 (0.02)
Some college	0.67 (0.02)***	0.45 (0.02)	0.66 (0.02)***	0.41 (0.03)	0.70 (0.02)***	0.53 (0.02)
≥College (ref)	0.90 (0.03)	0.72 (0.03)	0.79 (0.04)	0.60 (0.04)	1.08 (0.04)	0.93 (0.04)
100% fruit juice						
<High school	0.29 (0.02)*	0.13 (0.01)	0.37 (0.03)	0.18 (0.02)	0.22 (0.01)***	0.09 (0.01)
High school/equivalent	0.26 (0.01)**	0.09 (0.01)	0.24 (0.02)***	0.07 (0.01)	0.27 (0.02)*	0.12 (0.02)
Some college	0.37 (0.02)	0.16 (0.01)	0.39 (0.02)	0.16 (0.02)	0.33 (0.02)	0.16 (0.02)
≥College (ref)	0.35 (0.02)	0.14 (0.01)	0.38 (0.03)	0.15 (0.02)	0.33 (0.01)	0.14 (0.01)

Reference group identified in parentheses. Asterisks indicate significant difference in mean compared to the reference group (***p < 0.001; **0.001 < p < 0.01; *0.01 < p < 0.05).

## Discussion

The present analyses, based on a large nationally representative sample of US children and adults, show that the consumption of total fruit, measured in cup equivalents per day, fell far short of national goals [27]. Total consumption for the population ≥4y was just over 1 cup equivalent per day. In general, whole fruit contributed about 65% of all fruit servings, with 100% fruit juice contributing 35%, depending on age. The 2010 Dietary Guidelines for Americans recommended that no more than half of fruit servings be from 100% juice, while others have recommended limiting 100% fruit juice to one or two servings per day [1]. The present data suggest that, at the population-level, mean consumption levels of 100% fruit juice were well below these recommendations, as 9.2% of all participants age ≥ 4y consumed more than 1 serving of 100% fruit juice/d and 8.7% and 10.2% of children age 4-13y and 14-19y, respectively consumed more than 1 serving of 100% fruit juice/d. This work also shows that the consumption of whole fruit, as compared to 100% fruit juice, was strongly influenced by age, with young children consuming the most fruit juice and consumption falling rapidly with age. By contrast, the consumption of whole fruit showed a bimodal pattern with young children and older adults consuming the most.

Total fruit consumption varied across racial and economic groups. Generally, the failure to meet fruit recommendations was associated with being female, mid- to lower-income (particularly for adolescents and older adults), and being non-Hispanic black. By contrast, the highest total fruit consumption was associated with being male, higher income, and with graduate education (for adults). A similar socioeconomic gradient was obtained for the consumption of whole fruit. Lower-income groups and non-Hispanic blacks consumed the lowest amounts of whole fruit. The present data are consistent with previous findings from other representative population based-studies. Results from 1999-2008 NHANES show that participants in the Supplemental Nutrition Assistance Program (SNAP) consumed less whole fruit (0.7 servings vs. 1.1) but comparable amounts of 100% fruit juice (0.6 vs 0.5) compared to non-participants [17]. Despite the use of relatively crude dietary assessment tools, disparities in fruit consumption by income was also observed in data from the 2009 telephone-based Behavioral Risk Factor Surveillance System survey [32]. The same study also observed that 100% fruit juice made up a greater proportion of total fruit among the lowest-income respondents, consistent with the results of this study [32].

As noted, most published descriptive analyses of fruit do not disaggregate by types of fruit, and those that do tend to present the results for total fruit as the primary outcome. Given differences in the gradient of fruit consumption by race/ethnicity and SES, disaggregating fruits may be an important component of adequately measuring disparities in dietary intakes. For example, the Mexican-American population consumed significantly more total fruit than the non-Hispanic white population. However, this difference was driven by the greater consumption of 100% fruit juice (41% more) among Mexican-American’s compared to non-Hispanic whites. Similarly, for family income, there was limited evidence of a social gradient in total fruit, but strong evidence for whole fruit, an association that held upon adjustment for age, gender and race/ethnicity. Beyond dietary surveillance, disaggregation of measurements for 100% fruit juice from whole fruit may also play an important role in evaluating the efficacy of programs or interventions aimed at improving diet. Such disaggregation has been done for some dietary measures, notably the 2010 iteration of the Healthy Eating Index (HEI), which includes two scores for fruit: a total score (e.g., whole fruit and fruit juice) and a separate score for whole fruit [33].

In the present analyses, the greatest amounts of 100% fruit juice were consumed by young children, non-Hispanic blacks, and by lower-income groups. Those choices may be driven, in part by economic constraints. Observational data show that higher quality diets, including those that are higher in fruits and vegetables tend to be more costly than lower quality diets [34]. Data among adults from 2001–2002 NHANES show that among those consuming the highest cost diets, whole fruit consumption was close to optimum, whereas for those with the lowest cost diets, whole fruit consumption was 30% of recommended levels for men and 48% for women [34]. In a previous diet modeling study we found that replacing juice with comparable fresh fruit increased diet costs by about 13% [35]. However, replacing juice with lower-cost fruit (e.g., frozen and canned) increased costs by only 1.5%. Substituting juice with the three most commonly consumed fruits (oranges, apples, bananas) resulted in an increase in cost of 4%, suggesting that adding frequently available and lower-cost fruits to the diet may not result in large increases in diet costs.

Beyond potential economic constraints, there are additional challenges in increasing whole fruit consumption. One factor that might drive a preference for fruit juice over whole fruit includes the ease of storage, preparation and portioning, which may be particularly important in institutional settings, which are likely of particular importance to children’s dietary intakes [36]. Spoilage and wastage due to over-ripening of fresh fruit is also a challenge. According to USDA estimates, 25% of fresh fruit at the consumer level is lost due to over-ripening or spoilage, compared to 11% for processed fruits (which includes fruit juice along with canned/frozen fruit) [37]. Furthermore, juices may be more convenient for parents and caregivers who are likely operating under time constraints and look for easy and quick options [38, 39]. Beyond individual-level factors, there is some evidence that individuals residing in more deprived neighborhoods may have limited access to fresh fruits at local stores [40], which may influence fresh/whole fruit consumption [41]. In addition, while access to whole/fresh fruit in stores may vary by neighborhood characteristic and type of store, fruit juice is generally widely available when compared to whole fruit [42].

The study has important limitations. First, the NHANES data are based on self-report and are subject to random and systematic reporting errors. Unlike other foods, fruit consumption is not likely prone to systematic under-reporting given that it is generally regarded as a healthy food. Data on children, particularly younger children, is provided by a parent/guardian with knowledge of the child’s diet, which may result in reporting errors for fruit consumption. The study also had a number of strengths. First, the data are based on up to two non-consecutive 24-hour recalls which allowed for us to estimate the usual intakes of total fruit, whole fruit and fruit juice by socio-demographic group. Second, the availability of information on whole fruit and 100% fruit juice allowed us to disaggregate these two types of fruit. Failure to disaggregate these diverse foods makes it difficult to suggest program/policy solutions to improve dietary intakes. To date, few population-based studies have disaggregated fruits into whole vs. fruit juice, and many still report data from a combined fruit and vegetable definition.

### Conclusions & implications for practice

While current levels of fruit consumption fall short of national recommendations; at the population-level, 100% fruit juice consumption appears to be consumed at amounts less than or consistent with the Dietary Guidelines for Americans and American Academy of Pediatrics recommendations. If fruit juice were replace by whole fruit without a concomitant increase in whole fruit consumption, the shortfall in total fruit consumption will persist. Therefore, fruit juice consumption should continue to be monitored as efforts focus on increasing whole fruit consumption. This is important in light of dietary trends, where consumption of fruit juice appears to be decreasing over time, while whole fruit consumption is increasing [43]. In terms of dietary surveillance, given the observed differences in the socio-demographic trends in whole fruit and 100% fruit juice consumption, efforts to characterize the diet should disaggregate the types of fruit whenever feasible. Differences by income/education were most pronounced for whole fruit, suggesting that economic factors may play a role in explaining these differences. Applied research should evaluate reasons for differences in whole fruit consumption by socio-demographic group. Understanding the barriers and facilitators to fruit consumption is an essential step in determining the programs or interventions that are most likely to succeed in increasing fruit consumption. A number of behavioral and economic interventions have focused on increasing consumption of fruits, and also vegetables, by children, minorities, and low-income groups [44–46], though the long-term impact and scalability of these interventions has yet to be established. Effective interventions to improve diet quality require acknowledging and addressing behavioral, environmental and economic barriers and constraints.

### Availability of supporting data

All data used in the current research are publicly available from the National Center for Health Statistics. Data are available at: http://www.cdc.gov/nchs/nhanes/nhanes_questionnaires.htm.

## Abbreviations

NHANES: National Health and Nutrition Examination Survey
USDA: United States Department of Agriculture
MPED: MyPyramid Equivalents Database
NCI: National Cancer Institute
BRR: Balanced repeated replication
IPR: Income-to-poverty ratio.
